# Artificial Intelligence-Assisted Pathogen Detection: Algorithms, Biosensing Platforms, and Applications

**DOI:** 10.3390/bios16050267

**Published:** 2026-05-05

**Authors:** Jiani Liu, Wang Gao, Chengxi Guo, Wenzhuo Cai, Ziyan Tang, Song Li, Yan Deng, Xiaoguang Qu, Zhu Chen

**Affiliations:** 1 MOE Key Laboratory of Rare Pediatric Diseases, Hengyang Medical School, University of South China, Hengyang 421001, China; 2 State Key Laboratory of Advanced Fiber Materials, College of Materials Science and Engineering, Donghua University, Shanghai 201620, China; 3 Institute for Future Sciences, University of South China, Changsha 410008, China; 4 Department of Medical Oncology, The First Affiliated Hospital, Hengyang Medical School, University of South China, Hengyang 421001, China

**Keywords:** artificial intelligence, pathogen detection, machine learning, deep learning, point-of-care testing

## Abstract

Rapid and accurate pathogen detection serves as a core component in infectious disease prevention and control, clinical diagnosis and treatment, and public health surveillance systems. Although traditional detection methods have been widely adopted in clinical practice, they still exhibit significant limitations in terms of detection speed, throughput, automation levels, and adaptability to complex samples. In recent years, artificial intelligence (AI) technology has provided novel technical pathways for pathogen detection by leveraging its strengths in feature learning, pattern recognition, and multidimensional data modeling. The core contribution of this review lies in providing a novel, integrated analytical framework that overcomes the limitations of existing reviews, which often focus on a single modality (such as imaging alone or molecular diagnostics alone). Based on this framework, this paper systematically reviews AI research progress in pathogen detection, focusing on typical applications of machine learning and deep learning algorithms in analyzing imaging data, molecular diagnostic data, sensor signals, microscopic images, and multimodal data. It summarizes AI’s enabling value in enhancing detection sensitivity, specificity, automation, and point-of-care capabilities. Concurrently, this paper delves into key challenges facing AI-assisted pathogen detection, including data standardization, model generalization, interpretability, and clinical translation. It also outlines future trends toward intelligent, integrated, and clinically deployable applications. This paper aims to provide researchers and clinicians in the interdisciplinary field of artificial intelligence, biosensing, and clinical medicine with a comprehensive reference and roadmap for future development.

## 1. Introduction

Pathogens, including bacteria, viruses, fungi, parasites, mycoplasma, and other microorganisms, are the primary causative agents of infectious diseases in humans, animals, and plants. Accurate and timely pathogen detection is a cornerstone of clinical diagnosis, epidemic surveillance, and public health response systems, directly influencing early disease identification, therapeutic decision-making, and outbreak containment [1]. However, despite decades of technological advancement, conventional pathogen detection approaches still face substantial challenges in terms of sensitivity, speed, throughput, automation, and adaptability, particularly when dealing with complex biological matrices, low-abundance targets, or resource-limited settings [2].

Currently, widely used methods for direct pathogen detection include culture-based techniques, immunoassays, molecular diagnostics, and high-throughput analytical platforms [3]. The common goal of these technologies is to identify and characterize the pathogen itself or its specific biomarkers. Culture-based methods are regarded as the clinical “gold standard”, yet they are inherently time-consuming, often requiring hours to days, and are ineffective for fastidious or non-culturable pathogens. Immunological assays such as enzyme-linked immunosorbent assay and lateral flow tests offer simplicity and low cost, but their sensitivity and specificity are frequently insufficient for early-stage or low-load infections. Molecular diagnostic techniques, including polymerase chain reaction (PCR), loop-mediated isothermal amplification (LAMP), and clustered Regularly Interspaced Short Palindromic Repeats (CRISPR)-based assays, provide high sensitivity and specificity but rely on stringent reaction conditions, skilled personnel, and sophisticated instrumentation [4]. Meanwhile, high-throughput sequencing (NGS) and matrix-assisted laser desorption/ionization time-of-flight mass spectrometry enable broad-spectrum pathogen identification, yet their clinical deployment is constrained by high costs, complex data processing, and dependence on advanced bioinformatics expertise [5,6,7].

In addition to the limitations of their underlying technical principles, these traditional methods generally share a common bottleneck: inefficient extraction of clinically meaningful information from increasingly large and complex datasets—such as sequencing reads, mass spectra, microscopic images, and sensor signals [8]. Weak-positive signals, background noise, and cross-reactivity remain difficult to distinguish accurately [9,10], while diagnostic interpretation is often labor-intensive and subject to inter-operator variability [11]. Additionally, the growing diversity of sample types further complicates standardization and robust analytical performance across platforms.

These limitations have become particularly evident during recent global infectious disease outbreaks [12,13], including COVID-19, Ebola, highly pathogenic avian influenza (H5N1), and monkeypox [14,15,16,17], which are characterized by rapid transmission and large-scale dissemination. In such scenarios, delays or inaccuracies in diagnosis can significantly hinder effective disease control [18] and may compromise clinical outcomes due to reliance on subjective expert interpretation [19]. Consequently, there is an urgent need for innovative diagnostic strategies capable of delivering rapid, accurate, scalable, and automated pathogen detection [20]. In response, a growing body of research has explored novel platforms and analytical paradigms, including improved signal preprocessing pipelines, next-generation biosensing architectures, and data-driven diagnostic frameworks [20,21,22].

Among these emerging solutions, artificial intelligence (AI)—a branch of computer science dedicated to developing systems capable of simulating human intelligence, encompassing core functions such as perception, reasoning, learning, and decision-making [23,24,25]—has successfully revolutionized conventional laboratory testing and disease screening practices by automating diagnostic processes and processing large-scale, high-dimensional datasets [23,26]. demonstrating exceptional potential to address the inherent limitations of traditional pathogen detection technologies [27,28]. The value of AI unfolds along a clear and complementary clinical diagnostic pathway: at the level of direct pathogen detection, AI can be deeply integrated into next-generation biosensing architectures to achieve automatic, highly sensitive decoding and recognition of pathogen-specific signals [29]; at the level of infectious disease diagnosis, AI can drive data-driven diagnostic frameworks, assisting clinicians in rapid screening and diagnosis by analyzing infection-related pathological patterns in medical images (such as CT and X-rays), provide timely information to support clinical decision-making [30]. Although the latter does not directly identify pathogens, it provides critical clinical context and prioritization for pathogen diagnosis through intelligent interpretation of the consequences of infection. Together, these two elements—which are not in opposition—work in synergy to create a faster and more comprehensive diagnostic pathway.

The core strength of AI lies in its ability to automatically learn complex, nonlinear patterns from multidimensional data, enabling efficient mapping from raw signals to diagnostic outcomes. This capability encompasses intelligent signal decoding and automated interpretation, rapid classification and source tracing in complex samples, small-sample learning and transfer learning, as well as fully automated and integrated diagnostic pipelines [31,32,33,34,35]. These features directly address the critical shortcomings of conventional pathogen detection methods [30,36,37], offering faster and more reliable diagnostic outputs [38], reducing misdiagnosis and missed detection rates [39], and ultimately facilitating timely clinical intervention.

Motivated by the growing body of AI-enabled diagnostic innovations, this review provides a comprehensive synthesis of recent advances in artificial intelligence-assisted pathogen detection. Unlike studies that primarily focus on a single technological modality, this paper innovatively establishes a multimodal integrated analytical framework designed to provide a comprehensive overview of how AI empowers the entire process—from direct pathogen identification to the clinical diagnosis of infectious diseases. This integrated perspective helps reveal the complementarity and potential for convergence among different technological approaches. Specifically, we classified key AI algorithms according to data modalities such as imaging data, molecular diagnostic data, sensor signals, microscopic images, and multimodal datasets, and summarized their applications across these various data modalities. In addition, we clearly distinguished and examined the application of artificial intelligence in two key areas: pathogen identification and classification, and infection diagnosis and prognosis assessment. We focused on analyzing how AI enables rapid point-of-care testing and interpretable clinical decision support, while also carefully examining the challenges encountered in the process of clinical translation, including the reliability of gold standards, data heterogeneity, model interpretability, and regulatory considerations.

**Figure 1:**
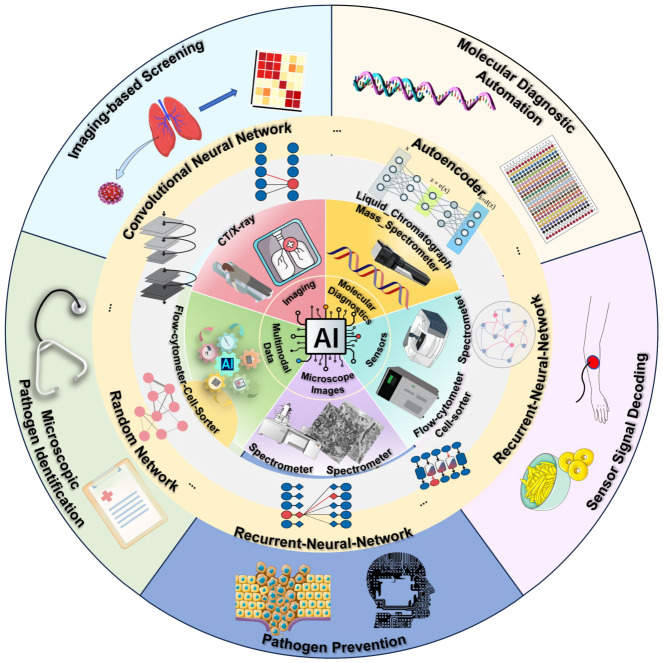
Schematic illustration of artificial intelligence-assisted pathogen detection and application scenarios.

Finally, future perspectives and developmental directions for AI-driven pathogen detection systems are proposed to facilitate their practical implementation in real-world clinical and public health settings (Figure 1).

## 2. Data-Centric AI Algorithms for Pathogen Detection and Diagnosis

The application of artificial intelligence in pathogen detection fundamentally involves the use of algorithms to learn patterns from data and to generate predictions or decisions. A clear understanding of the basic principles and applicability of core AI algorithms is, therefore, essential for grasping the development and future direction of this field.

In practical pathogen detection scenarios, the performance of algorithms is highly dependent on the modality of the input data. Data from different modalities—such as structured numerical data, images, spectra/signals, sequences, and text—possess unique characteristics and challenges, which in turn determine the computational paradigms and model architectures best suited for processing them. To address these challenges, various data-driven methods, including machine learning and deep learning, have been developed [25,39]. By automatically learning complex patterns from data, these methods support automated decision-making [40] and have been widely applied in multiple fields, including medical image analysis and electronic health record interpretation [23,38,39].

To systematically demonstrate how AI empowers different technical pathways, this section will organize representative algorithms and their applications based on the primary data modalities in pathogen detection: structured data, imaging data, spectral/signal data, and sequence data. The table below (Table 1) summarizes typical AI models across different modalities, along with their corresponding detection technologies, core advantages, and limitations. This section aims to establish a conceptual foundation to help readers better understand the subsequent discussions on AI-driven pathogen detection applications.

This table systematically reviews and compares the core characteristics, clinical performance trends (accuracy, sensitivity, specificity), strengths and limitations, and representative biomedical and pathogen detection applications of mainstream machine learning and deep learning algorithms across five primary data modalities: structured/tabular, image, sequence, spectral/signal, and multimodal. In terms of general performance, convolutional neural networks (CNNs) consistently achieve the highest diagnostic sensitivity and specificity in medical imaging and microscopic pathogen detection tasks [30,48,49], while traditional ensemble algorithms (Random Forest, XGBoost) and support vector machines (SVMs) deliver balanced accuracy and robustness for structured clinical data and small-sample pathogen classification [41,42]. Vision transformers (ViTs) exhibit superior global feature extraction capabilities but require substantially larger datasets to match CNN performance, and LSTMs remain optimal for capturing temporal patterns in genomic sequences and time-series signals [51,57]. Notably, the real-world robustness of all algorithms is frequently compromised by poor data quality, inter-laboratory variability, and inconsistent annotation standards, which remain a pervasive challenge in translational research. Crucially, no single algorithm universally outperforms others across all scenarios; the optimal choice must be tailored to the specific data characteristics, sample size, and clinical task objectives. Looking ahead, multimodal data fusion algorithms will emerge as the primary research direction to break the accuracy ceiling of single-modal detection, while enhancing model interpretability and reducing computational barriers remains a critical unresolved challenge.

## 3. AI-Enabled Pathogen Detection and Infectious Disease Diagnosis

Depending on the data sources and analytical objectives, the main technical approaches for AI-assisted diagnosis of infectious diseases and pathogen detection include infection-related imaging diagnosis, molecular pathogen detection, biosensor signal analysis, microscopic image recognition of pathogens, and multimodal data integration and analysis.

### 3.1. Imaging Diagnosis Related to Infection

Medical imaging techniques such as chest x-ray (CXR), computed tomography (CT), and magnetic resonance imaging (MRI) serve as vital tools for diagnosing pneumonia, tuberculosis, and other respiratory infectious diseases. Traditional radiological analysis heavily relies on radiologists’ experience for visual interpretation, with primary limitations including strong subjectivity, low reproducibility, significant dependence on specialized personnel, and difficulty in identifying subtle or atypical lesions [58,59].

The medical imaging techniques described in this section—such as CT and X-ray—primarily assist in the diagnosis of infectious diseases (such as pneumonia and tuberculosis) by identifying histopathological changes in the lungs, such as infiltrates, consolidation, and nodules, caused by pathogen infection. This is an auxiliary diagnostic method based on imaging findings that complements subsequent testing techniques designed to directly identify the pathogen itself.

**Figure 2:**
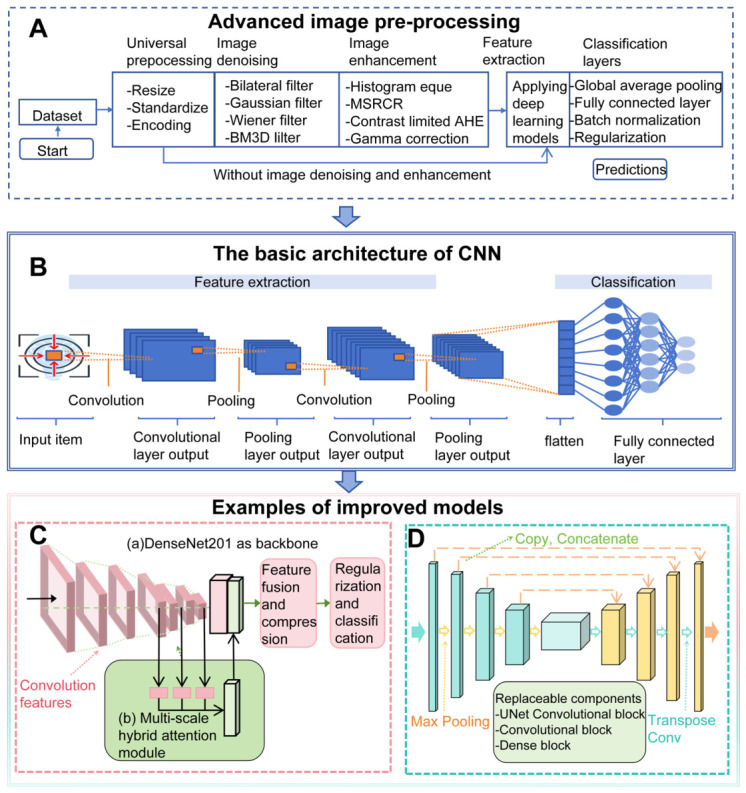

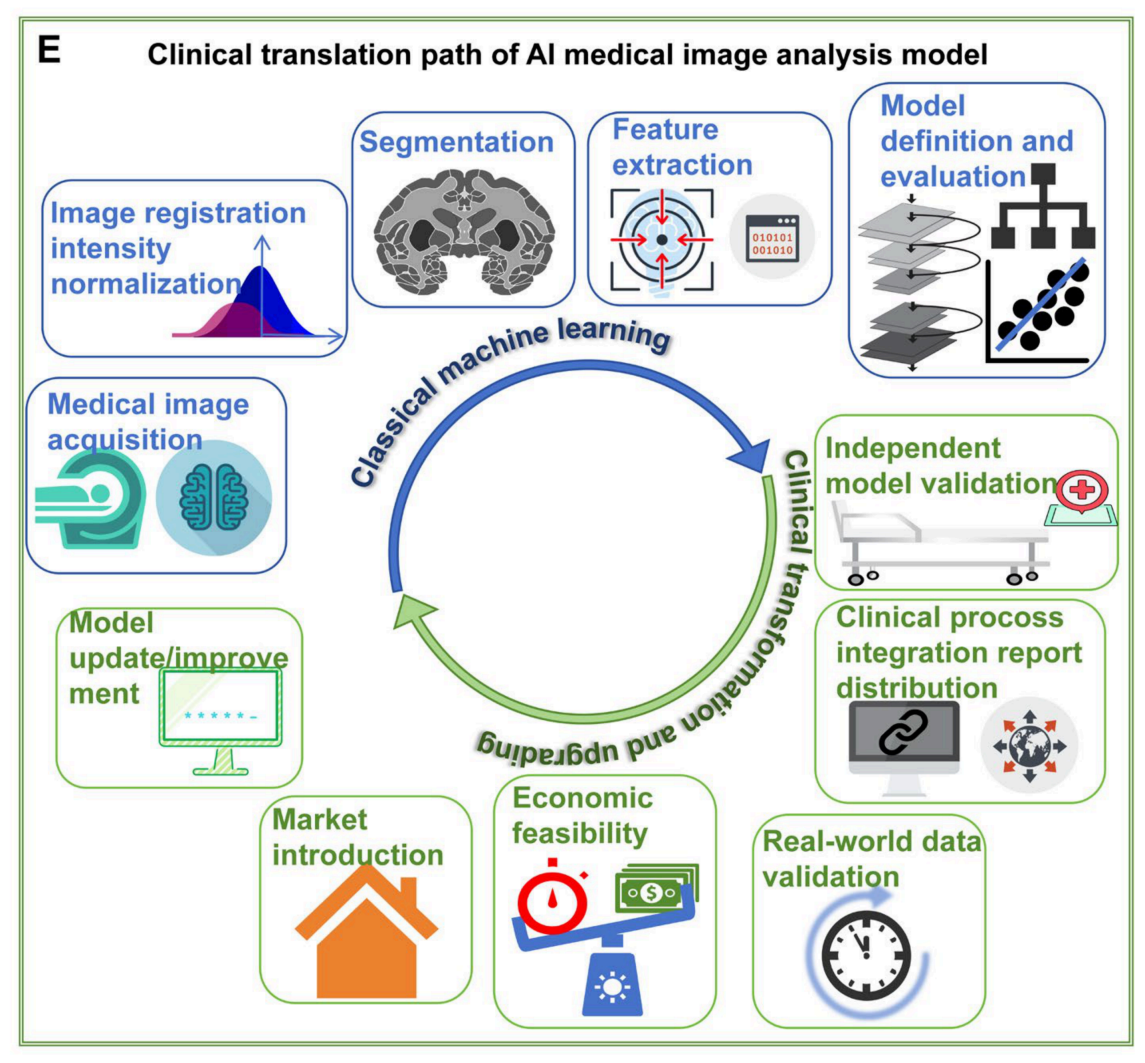
Development Pathways of Imaging Algorithms: (**A**) The basic architecture diagram of a Convolutional Neural Network (CNN); (**B**) The technical workflow diagram of image classification preprocessing, which lays the foundation for image recognition. The reconstruction of this workflow can be applied to model optimization and new application development; (**C**,**D**) The improved CNN structure; (**E**) The clinical translation path of an artificial-intelligence-based medical image analysis model.

In recent years, the introduction of AI technology has significantly improved the accuracy and efficiency of radiological diagnosis. DL models such as convolutional neural networks (CNNs) can automatically learn and extract deep texture, shape, and density features related to pathogen infection within images, thereby addressing the shortcomings of traditional methods [30,58,60,61,62]. Refined lesion identification can be achieved through model enhancements or image preprocessing, and the model can be iteratively refined through repeated clinical practice (Figure 2). For instance, the CNN-based Joint Foundation Chest X-ray 1 (JF CXR-1) software developed by Yang et al. demonstrated a sensitivity of 94.2%, specificity of 91.2%, and AUC = 0.98 in detecting pulmonary tuberculosis across 1161 subjects, with no serious adverse events observed [63]. The three-stage multi-task attention network COVID-MANet proposed by Sharma et al. enhances COVID-19 detection sensitivity and classification accuracy through lung region localization, the MA-DenseNet201 classification model, and the UNet infection segmentation module, combined with segmentation cropping and ensemble strategies [64]. As shown in Figure 2C, researchers improved the traditional “convolution-pooling-fully connected” CNN architecture by optimizing UNet and its derivatives for medical image segmentation tasks. The structure depicted in Figure 2D employs a “convolution + attention + fully connected” design to achieve end-to-end optimization of feature extraction, weight allocation, and output generation, significantly enhancing the model’s performance in fine-grained medical image recognition tasks. Figure 2E illustrates the full-cycle translation mechanism through which AI medical imaging models transition from the laboratory to clinical practice and are continuously upgraded and optimized based on real-world data and feedback.

In the field of chest x-ray applications, numerous studies have shown that artificial intelligence can effectively assist in the diagnosis of pneumonia and enable disease prediction. Borkowski et al. trained a model using the Microsoft CustomVision platform to classify COVID-19 pneumonia, non-COVID-19 pneumonia, and normal lung images, achieving 100% sensitivity, 95% specificity, and 97% accuracy [65]. Sharma et al. systematically evaluated the effectiveness of various image enhancement techniques combined with CNN models (e.g., MobileNet, DenseNet), finding that the time variant filter (TVF) + Gamma correction method achieved 93.25% accuracy in a four-classification task with 98.72% sensitivity for COVID-19 [66]. In CT applications, CT imaging combined with AI models can achieve diagnostic performance approaching human levels. An automated CT image analysis system developed by Yan et al. for tuberculosis detection and severity assessment demonstrated classification accuracy ranging from 81.08% to 91.05% on an independent test set, showing moderate to strong correlation with radiologist scores [67]. Qian et al. demonstrated that combining CT radiomics with clinical features can diagnose allergic bronchopulmonary aspergillosis (ABPA), achieving area under the curve (AUC) values of 0.896 and 0.886 on the training and test sets, respectively [68]. Wang and Shuai utilized an enhanced Inception model to analyze CT images, enabling COVID-19 clinical diagnosis prior to negative pathogen detection results [69]. In MRI applications, Wang et al. trained a convolutional block attention module using T2-weighted imaging and integrated it into the ResNeXt-50 architecture to develop a model distinguishing Brucella spondylitis from tuberculous spondylitis. Its accuracy, precision, recall, F1 score, and AUC outperformed ResNet50, GoogleNet, EfficientNetV2, and visual geometry group 16 [70]. We can also see how positron emission tomography-computed tomography (PET-CT) and ultrasound examinations are being integrated with AI. DL can identify lymph node metastasis features in PET-CT images with higher sensitivity than manual assessment, though specificity remains lower [71]. In breast ultrasound image analysis, AI achieves performance comparable to manual interpretation [72].

Although the aforementioned AI-driven medical imaging analysis technologies are not directly used for pathogen detection, they all reflect the pathological changes caused by infection, providing powerful, non-invasive tools for the clinical screening and diagnosis of infectious diseases. The integration of infection-related imaging diagnostic technologies with molecular diagnostics, biosensing technologies, and multimodal information—the latter of which can directly identify pathogens or their specific biomarkers (as discussed in detail below)—represents the future direction for achieving precise diagnosis.

### 3.2. Classification of Technologies Based on Molecular Diagnostics Data

Molecular diagnostic techniques (such as PCR, CRISPR, and metagenomic sequencing) enable precise pathogen identification by detecting nucleic acids, serving as the current “gold standard” for detecting various pathogens. However, traditional methods involve complex workflows, are time-consuming, and rely heavily on equipment and skilled operators, limiting their widespread adoption [73,74].

#### 3.2.1. AI-Enhanced Nucleic Acid Sequence Identification

In recent years, the integration of molecular diagnostics with AI has offered novel solutions for detecting unknown pathogens. For instance, the DCiPatho model developed by Jiang et al. combines k-mer frequency features with deep convolutional networks (DCNNs), outperforming existing methods across multiple performance metrics in 5-fold cross-validation. This approach significantly enhances the accuracy and generalization capability of pathogen identification within long genomic sequences [38].

#### 3.2.2. AI Optimization for Classical Nucleic Acid Detection Platforms

**Figure 3:**
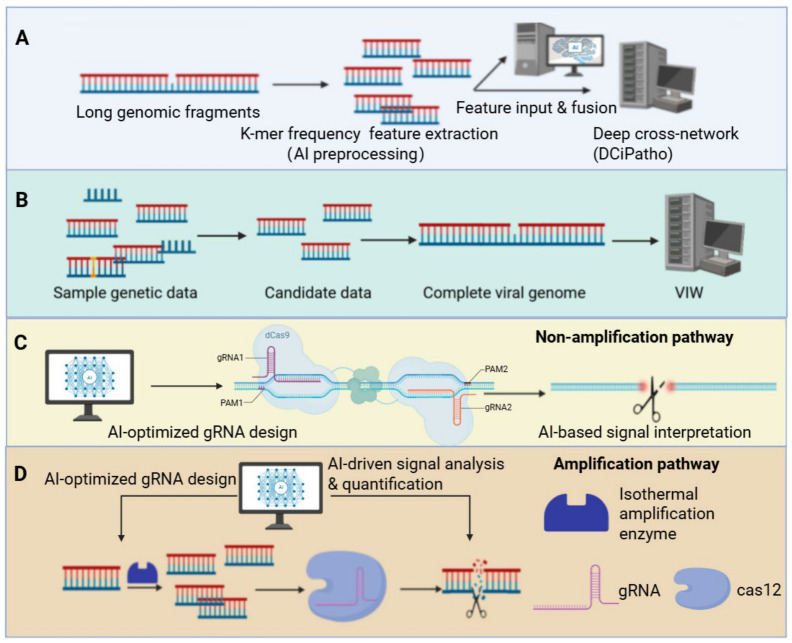
Application of AI-based molecular diagnosis in pathogen detection: (**A**) The process by which the DCiPatho model accurately identifies pathogens in long genomic sequences by combining k-mer frequency features with deep cross-network analysis; (**B**) The workflow diagram of metagenomic virus screening proposed by Song Shiyang; (**C**,**D**) Two AI-based methods for pathogen detection using the CRISPR system: (**C**) represents the non-amplification approach, while (**D**) depicts the amplification approach.

AI analyzes molecular-level pathogen data, primarily leveraging molecular information (e.g., pathogen genome sequences and nucleic acid fragments) obtained through synthetic biology tools such as the CRISPR system and Argonaute proteins. Algorithms interpret this molecular data to achieve precise pathogen identification [75,76,77,78,79,80] (Figure 3).

In virology applications, AI can optimize COVID-19 reverse transcription polymerase chain reaction (RT-PCR) testing by algorithmically refining reaction parameters and interpreting nucleic acid amplification signals, thereby reducing false positives and false negatives. Simultaneously, it enables rapid analysis of viral gene sequences (e.g., mutation sites) for precise pathogen subtype identification, enhancing detection efficiency and accuracy [30]. Furthermore, FENG et al. developed a “Standardized Swab Site Metadata Schema,” converting free-text swab site information (e.g., sampling location, environmental details) from open-source deoxyribonucleic acid (DNA) sequence databases into structured data comprising five information dimensions and 338 standardized terms. This schema directly empowers AI for pathogen molecular data analysis [81].

#### 3.2.3. AI-Driven High-Throughput Metagenomic Analysis

AI-assisted diagnostics also significantly enhance high-throughput sample processing capabilities. Song et al.’s metagenomic virus screening workflow completes the entire process from data quality control to viral genome reconstruction within minutes to hours, automatically generating visual reports that markedly improve emergency response efficiency during viral outbreaks [40,82]. The INSaFLU-TELEVIR platform developed by Santos et al. integrates multiple classification tools and databases to support rapid virus detection and phylogenetic analysis of real-time nanopore sequencing data. This effectively shortens the time from sample collection to diagnosis and enhances monitoring capabilities in clinical and public health laboratories [83].

#### 3.2.4. AI for Low-Abundance Pathogen Detection in Complex Samples

Furthermore, AI demonstrates advantages in detecting low-abundance pathogens. A systematic review by Roy et al. indicates that DL models (e.g., CNN, LSTM, autoencoders) excel at processing sparse, high-dimensional features in metagenomic data, achieving 10–25% higher F1 scores in sequence classification and disease prediction tasks, thereby enhancing the detection of low-abundance pathogens within complex microbial communities [84]. Overall, AI systems significantly enhance pathogen detection accuracy [85], propelling molecular diagnostics toward automation, intelligence, and personalization.

### 3.3. Classification of Technologies Based on Sensor Data

Sensor technologies detect pathogens by capturing physical or chemical signal changes (e.g., spectral shifts, color changes, electrical currents) generated when pathogens interact with specific probes. Traditional sensors rely on fixed thresholds or manual expertise to interpret signals such as spectral peaks, color intensity, or current strength. However, in complex biological samples, such methods are susceptible to background interference, non-specific binding, and weak signals, leading to reduced detection specificity and insufficient sensitivity [86]. Furthermore, signal interpretation varies across different sensor types, hindering standardization and unified analysis. Traditional approaches demonstrate limited capability in deciphering high-dimensional fluorescence signals from complex samples and addressing nonlinear interference between probes.

#### 3.3.1. Expansion of Sensor Types for Pathogen Detection

Beyond optical sensors, a variety of electrochemical, piezoelectric, and thermal sensors have been widely employed in pathogen detection. Electrochemical sensors, including amperometric, potentiometric, and impedimetric platforms, translate biorecognition events into measurable electrical signals and show unique advantages in miniaturization, low cost, and point-of-care applications. Piezoelectric sensors, such as quartz crystal microbalance (QCM), monitor pathogen-induced mass changes via resonant frequency shifts, while thermal sensors record enthalpy changes during specific binding processes. The introduction of AI enables unified analysis of diverse signal types, effectively overcoming the heterogeneity and complexity of data from different sensing modalities.

AI algorithms, particularly DL models, enable end-to-end learning and interpretation of high-dimensional, complex raw data generated by sensors [28,87]. AI does not rely on predefined thresholds but directly extracts deep information most relevant to pathogen type and concentration from raw data, significantly enhancing detection accuracy, interference resistance, and versatility. For instance, AI combined with Raman spectroscopy aids rapid detection of bacterial pathogens in clinical samples [88,89,90]. Usman et al. systematically reviewed recent advances in surface-enhanced Raman scattering (SERS) for bacterial pathogen identification, emphasizing the pivotal role of label-free SERS strategies integrated with AI DL in achieving rapid, highly sensitive clinical pathogen recognition [19,29,91]. The integration of SERS with machine learning for sensitive and selective detection of pathogens has also been demonstrated in nanobiosensor platforms [92].

#### 3.3.2. AI Application in Non-Optical Sensor Systems

For electrochemical sensors, machine learning models are capable of denoising voltammetric and impedance signals, distinguishing overlapping peaks, and improving quantitative accuracy in complex biological matrices. In piezoelectric and thermal sensing systems, AI algorithms effectively suppress non-specific adsorption and environmental interference, enabling reliable identification of trace pathogens.

AI also enables simultaneous detection of multiple pathogens and streamlined operations. Holliday et al.’s review highlights that machine learning-enabled colorimetric sensors can concurrently detect various foodborne pathogens with detection limits as low as 10^2^ CFU/mL. Results are obtained within 5 s via smartphone imaging and cloud-based AI, achieving non-destructive, multiplex detection in complex food matrices [93]. Hussain et al. employed spectral sensors (SERS, fluorescence spectroscopy) and microfluidic sensors, combined with AI analysis of pathogen optical signals (e.g., SERS RGB signals, fluorescence signals, and scattered light signals), to achieve rapid pathogen classification and concentration measurement. Representative examples include a smartphone-based SERS platform and a microfluidic fluorescence detection device for *E. coli* [94]. Related reviews systematically discuss the integration of AI with smartphones, confirming its advantages in rapid pathogen detection [95].

#### 3.3.3. Pathogen Identification and Classification Using Sensor Data

Regarding precise identification, AI significantly enhances the detection performance of sensor-based data. For instance, the colorimetric sensor developed by Materón Elsa M. et al., utilizing gold nanoparticles and smartphones, processes image color information via AI algorithms to achieve ultra-sensitive detection and 100% accurate diagnosis of Severe Acute Respiratory Syndrome Coronavirus 2 (SARS-CoV-2) in saliva [96]. Yi-Ming Tseng et al. integrated SERS with a vision transformer (ViT) model. Using SERS spectra from clinical blood samples, they achieved rapid bacterial identification with 99.30% accuracy for Gram staining, 97.56% accuracy for species classification, and 98.5% accuracy for methicillin-resistant *Staphylococcus aureus* (MRSA) detection, providing critical guidance for early sepsis treatment [97]. AI-enabled precision detection can advance sensor-based pathogen detection from general diagnostics toward personalized precision medicine.

#### 3.3.4. Accelerated Detection and Algorithm Optimization for Sensor Data

**Figure 4:**
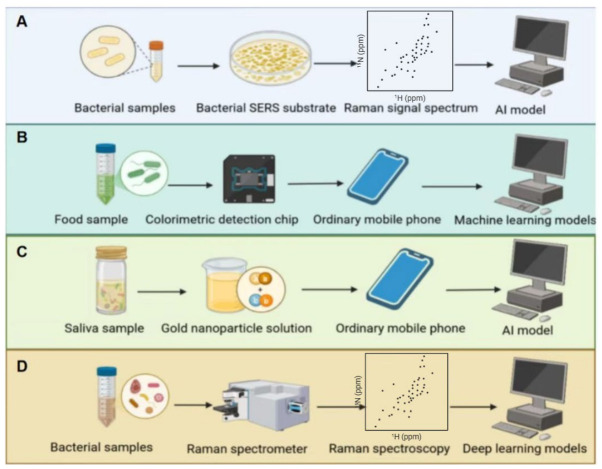
Application of AI-based sensor detection technology in pathogen detection: (**A**) Qiu Xun’s summary of the application of surface-enhanced Raman scattering (SERS) combined with artificial intelligence sensors for pathogen detection; (**B**) The principle of multiplex detection of foodborne pathogens by colorimetric sensors based on machine learning, comprehensively described by Holliday Emma G.; (**C**) A colorimetric sensing platform based on gold nanoparticles that integrates artificial intelligence for non-invasive pathogen detection in saliva samples. The “ordinary mobile phone” in the diagram refers to a consumer-grade smartphone used for image acquisition, which captures the colorimetric signal changes caused by the reaction between the saliva sample and the gold nanoparticle probe and finally enables intelligent identification of pathogens through an artificial intelligence model; (**D**) The workflow of high-precision pathogen detection by combining Raman spectroscopy with deep learning models, in which a professional Raman spectrometer is used to collect the characteristic Raman fingerprints of bacterial samples, and deep learning algorithms are applied to accurately classify and identify pathogens.

Furthermore, AI algorithm optimization shortens detection cycles while maintaining high accuracy. Liu et al. combined two-dimensional Raman spectroscopy with DL, converting spectra into images via wavelet packet transform and Gram angle field techniques. This approach significantly compressed data volume and reduced training time (by approximately 90%) while achieving 90.55% identification accuracy for 30 bacterial isolates [98]. Yan et al. developed a “CoFe_2_O_4_@HRP nanocomposite + dual-channel catalytic immunoassay strip” detection system for *E. coli* O157:H7, significantly enhancing detection accuracy and precision [99,100] (Figure 4). Figure 4C illustrates the detection principle developed by Materón et al., which employs a colorimetric sensor based on gold nanoparticles and a smartphone, utilizing AI algorithms to process image color information. Figure 4D demonstrates the principle of precise bacterial identification achieved by Liu et al. through the integration of wavelet packet transform and Gram angle field technology with DL.

#### 3.3.5. Explainable AI (XAI) for Bridging Model Output and Biological Mechanisms

Despite impressive performance, most AI-driven sensor models act as “black boxes,” lacking transparency regarding how decisions are made. Explainable artificial intelligence (XAI) methods, including SHAP, LIME, Grad-CAM, and attention mechanisms, have been introduced to bridge model outputs and underlying biological or biochemical mechanisms.

In optical sensing systems such as SERS and colorimetric sensors, SHAP and Grad-CAM identify key spectral bands or color features that dominate classification results, revealing which molecular vibrations or optical responses are responsible for pathogen identification. This confirms that the model relies on biologically meaningful signals rather than random noise. For electrochemical sensors, LIME and feature attribution decompose complex voltammograms into interpretable parameters such as peak current and potential, linking model outputs to specific redox reactions of pathogen-related biomolecules. In piezoelectric and thermal sensors, XAI quantifies the contribution of frequency shifts and enthalpy changes, helping distinguish specific binding from non-specific adsorption. By providing human-readable explanations, XAI not only improves the credibility and interpretability of detection results but also guides the rational design and optimization of sensing platforms.

#### 3.3.6. Complex Sample Analysis and Anti-Interference Capability Enhancement

The deep integration of artificial intelligence with nanobiological sensors has significantly advanced pathogen identification and multidrug resistance analysis [101]. For instance, Lin et al. [102]. combined multicolor DNA-silver nanoclusters (DNA-AgNCs) with highly conductive APBA-MXene nanomaterials to construct a bionic “taste” sensing system. This system employs convolutional neural networks (CNNs) for deep feature extraction of captured complex signal fingerprint spectra, enabling precise identification and quantitative analysis of single and mixed microbial communities in tap water with over 98.5% accuracy.

To address noise interference in real-world environments, Yi et al. [103]. developed a rapid, automated AI biosensing framework for pathogen detection in liquid foods and agricultural water. This model demonstrates strong generalization capabilities: despite being trained solely on pure laboratory cultures, it maintains 80–100% prediction accuracy when encountering real water samples with unknown noise while reducing detection cycles to 5.5 h.

#### 3.3.7. Agricultural and Hyperspectral Imaging-Based Pathogen Detection

Furthermore, integrating AI with hyperspectral or hyperspectral imaging technologies [104,105,106] enables the construction of efficient crop pathogen detection systems widely applicable in agricultural production. Applying AI to biosensors significantly enhances sensor performance, functionality, and real-time detection capabilities, thereby optimizing pathogen detection outcomes [85,107,108].

### 3.4. Technology Classification Based on Microscope Image Data

Microscopic imaging enables direct observation of pathogen morphology, motility, and staining characteristics, serving as a classic and fundamental method in microbiology. However, traditional microscopic analysis relies entirely on manual observation and counting by technicians through eyepieces, resulting in high labor intensity, low efficiency, limited throughput, and identification accuracy significantly influenced by operator experience, fatigue, and subjective judgment, making standardized and quantitative analysis challenging.

In addition to traditional and smartphone microscopes, AI has made groundbreaking progress in computational microscopy, overcoming the physical limitations of optics and enabling rapid, low-cost, and non-destructive pathogen detection without chemical labeling. AI virtual staining can digitally convert unlabeled images into equivalent H&E or Gram-stained images, eliminating the 1–2 h chemical staining process and avoiding sample damage [109]. A 2025 study in Science Advances showed that the virtual Gram staining generated by AI has accuracy comparable to traditional methods. In label-free imaging, AI can extract super-resolution information from low-contrast images such as phase contrast and quantitative phase images, enabling real-time identification of live pathogens. A 2026 study in PLOS Computational Biology demonstrated that visual Transformers achieve over 98% classification accuracy for seven common clinical pathogens.

DL-based computer vision technologies enable AI to automate the processing and analysis of microscope images, eliminating subjective variations caused by human error [110,111]. Trained models can rapidly scan digitized microscopic images, achieving fast, sensitive, and quantitative detection of target bacteria in complex backgrounds, offering new solutions for food safety and on-site pathogen detection [112,113]. For instance, in parasitology, Miri et al. employed convolutional neural networks (CNNs) to extract microscopic morphological features of Plasmodium parasites from blood smear images, distinguishing infected from uninfected red blood cells. In bacteriology, AI can automatically identify colony size, color, and other characteristics on culture media while counting colonies, replacing manual microscopy and significantly improving detection efficiency and consistency [30].

**Figure 5:**
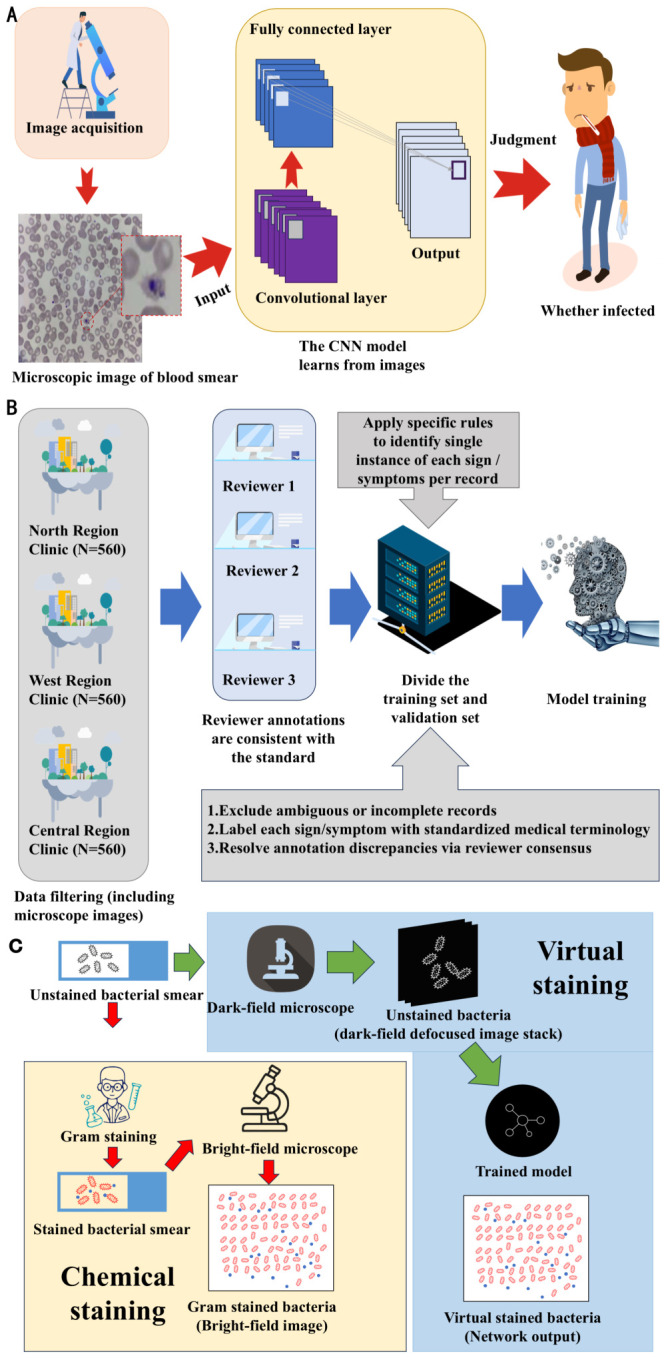
Flowchart of machine learning-based medical imaging diagnosis and analysis: (**A**) CNN-based blood smear microscopic image analysis workflow. DL automatically extracts Plasmodium falciparum morphological features to classify infected and non-infected red blood cells, enhancing detection objectivity and accuracy. (**B**) Multi-center clinical data standardization and evaluation workflow. Screening data is collected from multiple clinics, undergoes coding and performance assessment, and establishes a reference standard set to support standardized data management. (**C**) Bacterial samples are collected as defocused image sequences without staining using a dark-field microscope, then input into a trained deep learning model. This process can achieve a virtual staining effect simulating traditional Gram staining, without the need for chemical reagents or manual staining operations, supporting rapid observation of bacterial morphology, differentiation of Gram-negative/positive bacteria, and morphological analysis, covering the entire process from sample preparation, multi-focus image acquisition, model inference, to direct comparison with bright-field stained images.

Wang et al. developed the Clinical Histopathology Imaging Evaluation Foundation (CHIEF), trained on 60,530 whole slide images (WSIs) from 19 anatomical sites. Employing a two-stage pre-training strategy combining unsupervised tile-level feature extraction and weakly supervised whole-slide pattern recognition, coupled with attention-based feature aggregation and multi-task fine-tuning, CHIEF enables rapid, universal, and precise assessment of cancer detection, molecular profiling prediction, and survival prognosis in complex contexts. Compared to existing mainstream DL methods, overall performance improved by up to 36.1%, offering new avenues for digital pathology diagnosis and personalized cancer management [33] (Figure 5).

AI-assisted microscopy diagnostics significantly enhance detection objectivity, efficiency, throughput, and accuracy. Kim’s team combined 3D quantitative phase imaging with artificial neural networks to accurately identify 19 bloodstream infection-related bacteria from minimal bacterial cell samples, achieving 82.5% accuracy in single measurements and 99.9% after multiple measurements. Its performance rivals the mass spectrometry “gold standard,” providing an effective tool for ultra-early infection diagnosis [114]. Otherwise, AI substantially enhances detection sensitivity, pushing the performance limits of physical imaging. For instance, Pedro et al. analyzed optical microscope images on plasmonic substrates using SVM and MobileNetV3 algorithms, enabling detection of SARS-CoV-2 viruses as low as 1 PFU/mL—approximately 1000 times more sensitive than traditional Localized surface plasmon resonance (LSPR) sensing [115].

AI and Mobile Integration: AI can also be combined with smartphone microscopy to establish a “low-cost imaging-intelligent analysis-instant interpretation” detection system, overcoming the limitations of traditional pathogen detection that relies on specialized laboratories and lengthy cycles [116]. SHOKR A et al. developed the SPyDERMAN system using adversarial learning to assist mobile devices in processing microfluidic chip images, enabling precise detection of multiple viruses and viral nucleic acids [117]. These studies demonstrate AI’s broad application value in enhancing objectivity, efficiency, and accuracy within medical image analysis.

Overall, the deep integration of AI with microscopic imaging technologies enables the construction of rapid detection systems for relevant pathogens, further improving testing convenience and accuracy [118,119].

### 3.5. Technology Classification Based on Multimodal Data Fusion

In clinical practice, information derived from a single data source has inherent limitations, and unimodal AI algorithms face difficulties in simultaneously accommodating multidimensional clinical information for pathogen detection and disease diagnosis, exhibiting significant bottlenecks in generalizability and robustness. Therefore, multimodal data fusion has become a central research focus in this field. Building on existing studies, this paper deepens the methodological elucidation of multimodal fusion and supplements core technical details as well as the expansion of application boundaries.

**Figure 6:**
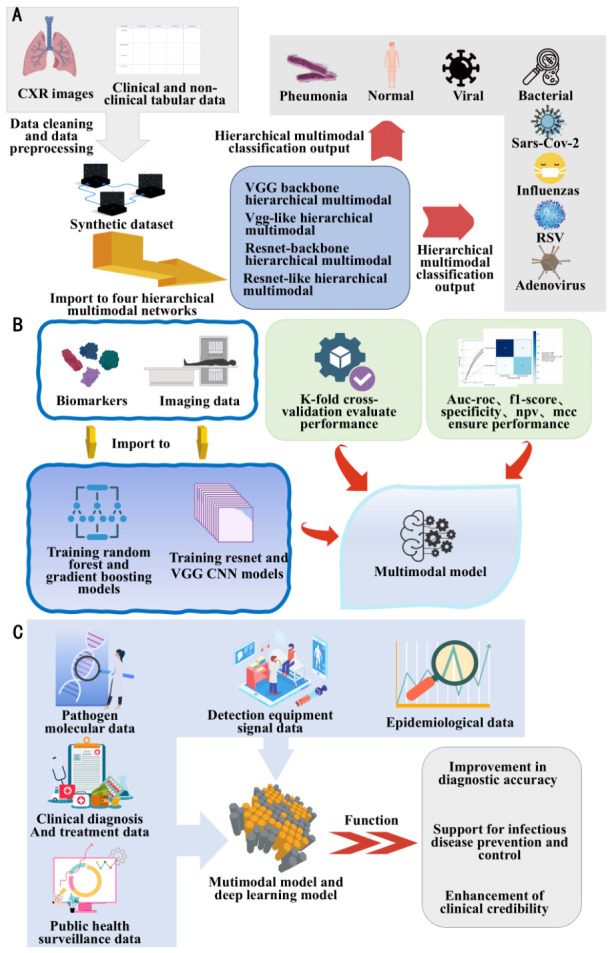
Flowchart of multimodal model-based intelligent diagnosis and application: (**A**) Multimodal lung disease analysis workflow encompassing high-dimensional data collection, preprocessing, synthetic dataset generation, feature extraction, and network construction. Achieves precise classification diagnosis of lung diseases through multimodal data fusion and DL. (**B**) Disease analysis modeling and validation using biomarkers and imaging data. Different models process corresponding datasets, employing K-fold cross-validation and multi-metric evaluation to enhance disease analysis accuracy and reliability. (**C**) Application logic of multimodal data fusion technology in pathogen detection. Integrating multi-source data with AI algorithms provides intelligent support for pathogen detection and related infectious disease prevention and control.

From the perspective of methodological core, multimodal fusion is mainly divided into two core strategies: feature-level (early) fusion and decision-level (late) fusion, which are adapted to different model architectures and clinical scenarios. Feature-level fusion first independently extracts features from each modality data, then integrates heterogeneous features into a unified feature vector input to the prediction model, maximizing the retention of original detailed information and suitable for various architectures such as tree models and CNNs. Li et al. [120], based on this strategy, integrated clinical features and CT radiomic features to construct a LightGBM multimodal model, whose core metrics, including AUC, accuracy, and sensitivity, all outperformed single-modality models. The external validation set achieved an accuracy of 0.745 and a sensitivity of 0.900, with performance superior to radiologists’ interpretations, NGS testing, and existing machine learning models (see Figure 6). Althenayan et al. [121] similarly adopted feature-level fusion, extracting CXR image features via pre-trained ResNet and VGG networks and integrating them with clinical data. The macro-average F1 score for the classification of eight lung diseases reached 95.9%, confirming the improvement in diagnostic accuracy achieved through multimodal fusion.

Decision-level fusion involves constructing independent prediction models for each modality of data, and then integrating the outputs of single-modality predictions to make decisions. This approach is highly compatible with heterogeneous data and can flexibly adapt the optimal algorithm for different modalities. Based on this strategy, Tur Kagan et al. [122] applied Random Forests and Gradient Boosting Machines to clinical biomarkers, and CNN to CXR images, then fused the results for further processing. The optimal Gradient Boosting–VGG combined model achieved an AUC-ROC of 0.94. At the same time, interpretability analyses using SHAP and LIME clearly identified the core decision features, further enhancing the clinical reliability and applicability of the model [123].

For the commonly encountered issues of modality missingness and sample imbalance in real clinical scenarios, existing mainstream solutions can be categorized into two core directions. To address modality missingness, generative models (GANs, VAEs) are often used for missing data imputation, adaptive attention weighting mechanisms are employed to reduce interference from missing modalities, or self-supervised contrastive learning is applied to learn cross-modal universal representations to enhance model robustness. To address sample imbalance, dynamic weighted loss functions, multimodal constrained resampling strategies, or federated learning for multi-center data aggregation are frequently adopted to mitigate model bias and improve generalization ability.

Beyond individual clinical diagnosis, multimodal fusion holds significant application value in epidemiological modeling and public health surveillance. Naumov et al. [124] developed a COVID-19 cloud platform that integrates multimodal data, including viral genomics, blood biochemistry, clinical pathology, and transcriptomics. Using a LightGBM-based model (F1 score 0.77), they successfully identified key viral mutations and risk factors, providing decision support for epidemic prevention and control. Building on this, the technology can be further extended to scenarios such as early warning of infectious disease outbreaks (integrating multidimensional data such as pathogen monitoring, clinical visits, and population mobility), pathogen tracing and transmission chain analysis, and stratification of population infection risk, offering end-to-end technical support for the optimization of public health prevention strategies.

In summary, through the optimization of core strategies, breakthroughs in technical bottlenecks, and expansion of application scenarios, multimodal data fusion can not only enhance the performance of clinical pathogen detection and disease diagnosis but also provide crucial support for public health infectious disease control. It represents a core future development direction in this field.

Overall, the integration of multimodal data fusion with AI represents a trend toward more comprehensive, reliable, and precise auxiliary diagnostic technologies. This approach not only enhances the accuracy of pathogen detection and disease diagnosis but also provides crucial technical support for early warning, source tracing analysis, and personalized treatment.

### 3.6. Clinical Translation of Point-of-Care Testing and Interpretable AI

This section serves as a clinical-level summary of the technical details discussed in Section 3, focusing on the practicality of AI implementation and the building of trust; it does not repeat descriptions of specific algorithms or hardware architectures.

AI-enabled point-of-care testing decentralizes diagnostic decision-making to communities, primary care clinics, and patients themselves. Its applications are inclusive, particularly in resource-limited settings such as remote areas and emergency rooms. By simplifying operational workflows (e.g., AI-enabled readers and voice guidance) and recalibrating algorithms to account for environmental variability, AI has enabled natural language interfaces tailored to low-literacy settings, allowing non-professional users to obtain reliable results. This marks a shift from “technological superiority” to “universal accessibility” [125].

Explainable AI serves as a bridge between high-precision models and clinical trust, offering both humanistic and decision-making value. In pathogen identification tasks, explainable AI assists laboratory personnel in verifying results by visualizing key nucleic acid sites or antigenic epitopes, thereby reducing concerns about “black-box” models. In clinical diagnosis tasks, explainable AI helps physicians understand the AI’s “reasoning process” by annotating suspicious lesions in images with heatmaps or indicating risk probabilities, enabling them to make more confident intervention decisions in fast-paced clinical settings [126].

In summary, the clinical implementation of AI depends not only on sensitivity but also on design tailored to specific clinical scenarios and transparent explanations of decision-making—a critical step in bringing the technology from the laboratory to the patient’s bedside.

## 4. Conclusions

By deeply integrating into pathogen detection processes—including imaging, molecular diagnostics, spectroscopy, microscopic image analysis, and multimodal data fusion—artificial intelligence technology offers novel pathways for pathogen detection. Significant breakthroughs have been achieved in enhancing efficiency, improving detection accuracy, optimizing pathogen surveillance and public health control [127], advancing personalized precision medicine, and boosting point-of-care testing capabilities. Simultaneously, AI applications reduce reliance on human labor and equipment, demonstrating unique advantages and broad application prospects across multiple medical fields.

Nevertheless, widespread adoption of AI in routine clinical practice still faces numerous challenges [14]. First, model performance heavily depends on large-scale, high-quality, and accurately annotated datasets. Barriers exist in acquiring, standardizing, and protecting medical data privacy. Balancing accelerated diagnosis and treatment with ethical safeguards and patient safety remains an unresolved issue [128]. Second, the issue of model interpretability (“black box” phenomenon) limits clinicians’ full trust and adoption. More interpretability tools (such as SHAP and LIME, used in Tur Kagan’s research) are needed to clarify the basis for decision-making [122]. Furthermore, integrating and validating AI tools in clinical settings is complex. Seamless embedding into existing workflows requires rigorous multicenter clinical trials to demonstrate efficacy and cost-effectiveness, with clinical acceptance and implementation costs remaining significant barriers [129]. Observational studies can evaluate the real-world impact of AI-assisted systems, analyzing relationships between usage patterns, user demographics, geographic regions, and cost-effectiveness to inform model refinement [130].

However, it should be noted that the evaluation of AI model performance relies heavily on the quality of the “gold standard.” In the field of pathogen detection, commonly used methods such as culture, PCR, and sequencing inherently have limitations in terms of sensitivity and specificity, as well as delays in results or blind spots regarding unknown pathogens. The inherent variability and imperfections of these “gold standards” are directly “carried over” to AI models, affecting their training effectiveness and reporting performance [131]. In the future, more reliable benchmark datasets and evaluation protocols need to be developed.

With regard to the development of artificial intelligence itself, we believe it requires continuous breakthroughs at both the technological and practical levels. Technologically, more efficient, lightweight, interpretable, and user-friendly AI models should be developed, with enhanced multimodal fusion research to deliver tangible benefits for patients and clinicians by reducing costs, shortening diagnostic cycles, and minimizing errors. Practically, cross-institutional and cross-regional data-sharing and collaboration platforms should be established to promote the joint creation and sharing of high-quality datasets; rigorous standards and regulations must be established to ensure the safety, efficacy, and fairness of AI tools; and training for healthcare professionals in AI applications should be enhanced to guarantee sustainable clinical implementation. Through interdisciplinary collaboration, continuous technological innovation, improved policy safeguards, and effective AI system deployment, artificial intelligence holds the potential to play a pivotal role in building a more resilient global public health defense system, enabling precision, efficiency, and universal access in healthcare practices.

## Figures and Tables

**Table 1:** Comparison of Different Artificial Intelligence Algorithms in Pathogen Detection.

Data Modality	Representative Model	Applicable Pathogen Types	Sensing Mode	Core Features	Advantages	Limitations	Typical Application Scenarios
Structured /tabular data	Random forest, XGBoost [41]	Bacteria (*Escherichia coli*, *Salmonella*, *Staphylococcus aureus*), fungi, viruses, parasites	Spectral sensing (Raman, infrared, fluorescence spectra); electrochemical sensing; biosensor array signals	Multi-model ensemble, multi-tree voting	Resistant to overfitting, capable of handling high-dimensional features	The model has poor interpretability and requires substantial computational resources.	Multimodal Data Fusion, Pathogen Classification [42]; Diagnostic Prediction of Mycoplasma Pneumoniae Pneumonia in Children [43]
	Gradient boosting	Foodborne pathogens, respiratory viruses, aquaculture pathogens	Electrochemical impedance spectroscopy; optical biosensing; microfluidic sensing signals; image sensing (colony images); simple optical sensing; portable rapid detection sensors	Gradually correct errors, enhance predictive capabilities	High precision, strong flexibility	Long training time, prone to overfitting, requires parameter tuning.	Rapid detection of pathogenic bacteria and their mixtures in water and milk [44]
	K-nearest neighbor (KNN) [45]	Common bacteria, fungal spores, and simple viruses	Electrochemical impedance spectroscopy; optical biosensing; microfluidic sensing signals; image sensing (colony images); simple optical sensing; portable rapid detection sensors	By calculating the distance between the sample to be predicted and the training samples, the majority class or mean of the K nearest neighbors is taken as the result	Requires no training process, adapts quickly to new data, handles multi-class classification problems, and is easy to implement.	Highly sensitive to the “curse of dimensionality” in high-dimensional data, computationally intensive, and dependent on distance metric selection.	Rapid identification of *Escherichia coli* O157: H7 and listeria monocytogenes in dairy products [46]
Image data	Convolutional neural network (CNN) [47]	Bacterial colonies, fungi, parasite eggs, virus microscopic images	Microscopic image sensing (bright-field, fluorescence, confocal); spectral imaging sensing; colony image identification	Automatic extraction of image features	Demonstrates outstanding performance with image data, enabling end-to-end learning	Requires a large amount of annotated data and significant computational resources	Tuberculosis screening; Chest x-ray and computed tomography (CT) imaging analysis [48]; Determine whether red blood cells are infected with malaria parasites [30]; Detection and identification of pathogens causing prosthetic joint infection [49]; Detection of target bacteria by the modified M13 bacteriophage [33]
	Vision transformer (ViT) [50]	Complex morphological pathogens, high-resolution microscopic pathogens, multiple mixed infections	High-resolution microscopic imaging; wide-field imaging, digital pathology images; multimodal visual sensing	Image processing based on Self-Attention Mechanism	Supports parallel computing with strong global feature capture capabilities	Data requirements are extremely high, and computational costs are significant	Analysis of bacterial Gram type using Raman spectroscopy image analysis [51]
	Generative adversarial Network (GAN) [45]	Data-scarce pathogens, hard-to-culture pathogenic bacteria, rare viruses	Sensor data enhancement (spectral, image); small-sample microscopic images; noise sensor signal restoration	The adversarial system consists of a generator and a discriminator. The generator produces simulated data, while the discriminator distinguishes between real and simulated data. Through adversarial training, the model is optimized	The generated data exhibits high authenticity and diversity, enabling unsupervised/semi-supervised learning and supporting data augmentation	Training process instability (prone to mode collapse), difficulty in determining model convergence, and poor interpretability of generated results	Detecting cells in cross-modal Images using GANs [52]
Spectral/Signal Data	Autoencoder [53]	Universal for all pathogens, especially high-dimensional spectral detection	High-dimensional spectral sensing (infrared, Raman, near-infrared); sensing signal denoising, feature extraction; multimodal biosensing fusion	Unsupervised learning, used for feature dimensionality reduction or generation	Capable of processing unlabeled data with strong feature extraction capabilities	May learn irrelevant features, resulting in poor interpretability	High-speed diagnosis of bacterial pathogens at the single-cell level through integration with Raman microscopy and machine learning filters [54]
Sequence Data	Recurrent neural network(RNN)/long short-term memory (LSTM) [48,55,56]	Dynamic bacterial growth, real-time pathogen monitoring, continuous cultivation of pathogenic bacteria	Temporal electrochemical sensing; real-time fluorescence monitoring; dynamic impedance sensing; continuous online biosensing	Processing sequence data based on temporal dependencies with memory capabilities	Suitable for genomic sequence and time-series signal analysis, capable of capturing dynamic evolutionary features	The training process is prone to gradient vanishing and exhibits low efficiency in processing long sequence data	Identification of unknown pathogens in metagenomic data tracking viral variation trajectories in wastewater samples [57]

## Data Availability

All data discussed are derived from published studies cited in the references, which are accessible via standard academic databases.

## Abbreviations

The following abbreviations are used in this manuscript:

AI	Artificial Intelligence
PCR	Polymerase chain reaction
LAMP	Loop-mediated isothermal amplification
CRISPR	Clustered regularly interspaced short palindromic repeats
NGS	High-throughput sequencing
NLP	Natural language processing
SVM	Support vector machine
KNN	K-nearest neighbor
CNN	Convolutional neural network
DL	Deep learning
CT	Computed tomography
ViT	Vision transformer
RNN	Recurrent neural network
LSTM	Long short-term memory
CEMRI	Contrast-enhanced magnetic resonance imaging
GAN	Generative adversarial network
CXR	Chest x-ray
MRI	Magnetic resonance imaging
JF CXR-1	Joint Foundation Chest X-ray 1
TVF	Time variant filter
ABPA	Allergic bronchopulmonary aspergillosis
AUC	Area under the curve
PET-CT	Positron emission tomography-computed tomography
RT-PCR	Reverse transcription polymerase chain reaction
DNA	Deoxyribonucleic acid
SERS	Surface-enhanced Raman scattering
MRSA	Methicillin-resistant *Staphylococcus aureus*
CHIEF	Clinical Histopathology Imaging Evaluation Foundation
WSIs	Whole slide images
LSPR	Localized surface plasmon resonance
AUC-ROC	Area under the receiver operating characteristic curve
SHAP	SHapley Additive exPlanations
LIME	Local interpretable model-agnostic explanations
GNN	Graph neural network
GBDT	Gradient boosting decision tree
